# Screening, Diagnostic and Prognostic Tests for COVID-19: A Comprehensive Review

**DOI:** 10.3390/life11060561

**Published:** 2021-06-14

**Authors:** Mariana Ulinici, Serghei Covantev, James Wingfield-Digby, Apostolos Beloukas, Alexander G. Mathioudakis, Alexandru Corlateanu

**Affiliations:** 1 Department of Preventive Medicine, Discipline Microbiology and Immunology, State University of Medicine and Pharmacy “Nicolae Testemitanu”, 2004 Chisinau, Moldova; mariana.ulinici@usmf.md; 2 Department of Respiratory Medicine, State University of Medicine and Pharmacy “Nicolae Testemitanu”, 2004 Chisinau, Moldova; kovantsev.s.d@gmail.com; 3 Division of Infection, Immunity and Respiratory Medicine, School of Biological Sciences, The University of Manchester, Manchester Academic Health Science Centre, Manchester M23 9LT, UK; James.Digby@mft.nhs.uk (J.W.-D.); alexander.mathioudakis@manchester.ac.uk (A.G.M.); 4 The North West Lung Centre, Wythenshawe Hospital, Manchester University NHS Foundation Trust, Manchester M23 9LT, UK; 5 Department of Biomedical Sciences, University of West Attica, 12243 Athens, Greece; 6 Institute of Infection & Global Health, University of Liverpool, Liverpool L69 7BE, UK

**Keywords:** SARS-CoV2, COVID-19, coronavirus, diagnosis, screening, prognosis, PCR, CRISP, immunoglobulin

## Abstract

While molecular testing with real-time polymerase chain reaction (RT-PCR) remains the gold-standard test for COVID-19 diagnosis and screening, more rapid or affordable molecular and antigen testing options have been developed. More affordable, point-of-care antigen testing, despite being less sensitive compared to molecular assays, might be preferable for wider screening initiatives. Simple laboratory, imaging and clinical parameters could facilitate prognostication and triage. This comprehensive review summarises current evidence on the diagnostic, screening and prognostic tests for COVID-19.

## 1. Introduction

Declared a pandemic in March 2019, COVID-19, which is caused by the novel severe acute respiratory coronavirus 2 (SARS-CoV-2) has caused not only significant global morbidity (>160 million cases) and mortality (>3.4 million deaths), but also disruption to society and economies [1,2,3,4]. The true incidence of COVID-19 is likely to have been underestimated, either due to individuals underreporting mild symptoms or inadequate testing. Screening and diagnostic strategies for COVID-19 have varied worldwide according to government policies, technology, funding, and data management capabilities. Even in industrialised nations, uncertainties in strategy, regulatory hurdles and supply issues have disrupted testing capabilities, possibly contributing to worsening spread of SARS-CoV2. Standardisation and improvement of COVID-19 diagnostics, with more efficient detection and treatment of cases, is likely to be beneficial to both industrialised and low-income nations [5,6,7]. In this review, we address some of the key concerns regarding laboratory and radiological screening, diagnosis and prognostication that have arisen during the ongoing COVID-19 pandemic [8]. A detailed description of the characteristics of the available screening and diagnostic assays and medical devices is beyond the scope of this manuscript and can be found in the Joint Research Centre of the European Medicines Agency/European Commission (https://covid-19-diagnostics.jrc.ec.europa.eu/devices, accessed on 10 June 2021).

## 2. Diagnostic Testing

Confirming diagnosis, starting treatment (where necessary) and initiation of social-distancing measures are essential in the management of COVID-19. Numerous methods are utilised for the laboratory diagnosis of viral infections, such as culture, viral nucleic acid detection and serology (i.e., viral antigen or humoral responses) [9]. The most commonly available tests are direct (detection of nucleic acids (i.e., viral genome) or antigens of the virus) and indirect (assessment of serum antibody levels).

### 2.1. Direct Testing—Molecular Methods

Viral nucleic acid detection, directly detecting genetic material, is the gold standard in diagnostic virology [9]. Two commonly preferred nucleic acid-based detection methodologies are available for the diagnosis of SARS-CoV-2. RT-PCR based methods (real-time polymerase chain reaction (RT-PCR)) are routinely used for diagnosis, while high throughput genome sequencing is used at an unprecedented rate for COVID-19 variants surveillance [10].

RT-PCR has frequently been utilised in the detection of COVID-19 nucleic acids from a number of sources such as posterior oropharyngeal saliva, throat and nasopharyngeal swabs, sputum and bronchial fluid [11]. Moreover, in some cases the virus can be isolated from blood, semen and faeces, even in the absence of a positive respiratory sample [12]. However, a recent systematic review concluded that RT-PCR tests from stool, urine, and plasma were less sensitive than respiratory samples [13].

In spite of RT-PCR being the benchmark for COVID-19 diagnosis, there is a potential for false-negative results, which may be related to the viral load, sampling time or sampling bias. RT-PCR tests typically provide a positive result if the specimen is collected 2–8 days after the onset of the infection [14]. A meta-analysis, which assessed the accuracy of COVID-19 tests and included 34 studies with 12,057 COVID-19 confirmed cases reported false-negative RT-PCR rates between 2% and 29%. The reported sensitivity was 71-98%, based on individuals with an initial negative RT-PCR being subsequently found to have a positive one. COVID-19 diagnostic accuracy may be improved by combining results of RT-PCR, imaging and serology screening [13].

Several other methods, namely, droplet digital PCR (ddPCR) and reverse transcription-loop-mediated isothermal amplification (RT-LAMP), which detect SARS-CoV-2 RNA are being also used to complement RT-PCR. Overall, ddPCR performs better than standard RT-PCR for clinical diagnosis of SARS-CoV-2, reducing false-negative results, although it is less thoroughly evaluated compared to the latter [15,16]. This is further highlighted by evidence showing the ddPCR-based can effectively detect SARS-CoV-2 genome in symptomatic cases with a negative standard RT-PCR [17]. A recently published study reported that combining RT-PCR with ddPCR improves the sensitivity from 40% (95% CI: 27–55%) to 94% (95% CI: 83–99%) without limiting the specificity, which remains 100% (95% CI: 48–100%), leading to an overall increase in the diagnostic accuracy from 47% (95% CI: 33–60%) to 95% (95% CI: 84–99%) [16].

Rapid diagnosis of COVID-19 is important, and some methods provide a result in less than an hour. RT-LAMP detects SARS-CoV-2 RNA in approximately 30 min with good correlation to the conventional RT-PCR, allowing for rapid delivery of results to patients both in the hospital and community settings [18]. RT-LAMP has been reported to have high sensitivity and specificity, operational simplicity and low cost [19].

Data on analytical performance (i.e., sensitivity, specificity, lower limits of detection (LOD), etc.) for all commercially available molecular assays are provided by FIND (a global alliance for diagnostics, which seeks to ensure equitable access to reliable diagnosis around the world) and can be assessed in the following directory, which is regularly updated (https://www.finddx.org/test-directory/, accessed on 10 June 2021).

Alternative approaches based on combinations of isothermal amplification and clustered regularly interspaced short palindromic repeats (CRISPR) such as the SHERLOCK (specific high-sensitivity enzymatic reporter unlocking) technique reduce dependence on RT-qPCR equipment [20]. SHERLOCK was found to be 98.5% specific and 93.1% sensitive with a lateral-flow readout [20]. The fluorescence readout was 100% specific and 96% sensitive for the full range of viral load in the clinical samples. In 380 SARS-CoV-2-negative samples obtained prior to surgery, SHERLOCK matched quantitative PCR with reverse transcription in 100% of cases [21,22]. However, data on RT-LAMP and CRISPR are significantly more limited than on PCR methods. Further validation is required. A comparison of the molecular-based direct diagnostic methods is shown in Table 1.

Efficient testing in COVID-19 is important but the time in which results are generated varies significantly, not only with choice of molecular tests, but also due to an array of factors not limited to transportation, processing and reporting [8]. Speed of results could certainly be improved with better test-to-laboratory and results to clinician pathways, but improving the efficiency of these steps is not always straightforward. The COVID-19 pandemic has however highlighted the importance of low-cost rapid point of care (POC) virology diagnostics [19].

Of the hundreds of SARS-CoV-2 diagnostic test assays under development or in use, syndromic testing is of particular interest in tackling the pandemic. This is the process of simultaneously targeting and detecting multiple pathogens that cause similar signs and symptoms, especially during the winter season when multiple different viruses could be circulating.

Syndromic respiratory testing, such as the Multiplex RT-PCR tests that detect multiple respiratory viruses in the same specimen including SARS-CoV-2, influenza, and 21 other common respiratory viral and bacterial pathogens have huge potential to streamline diagnosis and early disease specific treatment [23,24]. An added benefit is the speed in which syndromic tests return.

There is still a role for single COVID-19 tests, when pre-test probability is high or contact tracing is required. On the over hand, syndromic tests are extremely useful when symptoms are non-specific, and the list of possible diagnoses is long. Similarly, diagnostic accuracy is more important in patients with moderate or severe disease, as SARS-CoV2 management (dexamethasone, anti-virals, immunomodulatory treatments), differs significantly to that of alternative conditions.

### 2.2. Direct Testing—Antigen Testing

Antigen tests can detect the presence of SARS-CoV-2 viral proteins in nasal or pharyngeal swabs and saliva. Most are lateral-flow chromatographic immunoassays (point of care, rapid detection) or chemiluminescence immunoassays (laboratory-based). Although direct antigen tests are increasingly used, the diagnostic accuracy of these tests is lower in comparison with that of RT-PCR [25]. There are some concerns regarding their specificity, since they may cross-react with other coronaviruses, but the main issue is their sensitivity [26]. The sensitivity of such panels ranges between 34% and 88%, based on the findings of a Cochrane systematic review [25,27]. Therefore, despite the higher accuracy of positive results, negative results should be treated more cautiously. Their diagnostic accuracy is greatest in symptomatic patients, when the viral load in the nasopharynx is higher, enabling early diagnosis, triage and treatment [28]. The optimum timing of testing can vary (within 5, 7 and 12 days from symptom onset) [29]. Therefore, the advantages of point of care tests, with rapid identification and isolation of cases, at a lower cost to RT-PCR tests, may perhaps be limited to some extent by the poor sensitivity they offer, especially in asymptomatic or pre-symptomatic individuals. Despite these limitations, the main advantages of antigen tests are speed (usually around 5–30 min compared with hours for PCR-based techniques), ease of interpretation and the limited technical skill and infrastructure required. This is particularly important for resource-limited settings [30]. In balance, it has been proposed that rapid and real-time viral antigen detection assays have been shown to be more useful in controlling the spread of the infection than more sensitive, but more expensive tests with more prolonged test time, such as the molecular tests [14].

### 2.3. Indirect Testing

Serological testing is an alternative means of diagnosing COVID-19. Whereas in most infections the IgM response precedes IgG, the distinction in COVID-19 (where both can be detected in the initial weeks) is less clear. This explains the similarities in the pooled sensitivity of both IgM and IgG in the early weeks of COVID-19 infection—33% and 23% at one week and 73% and 68% at two weeks, respectively [31]. At three to four weeks the picture begins to change, with IgG or total antibody tests providing the greatest sensitivity (low false-negative results) and specificity (low false-positive results) compared to other antibody types [31].

Several methods have been proposed for accurate assessment of IgG and IgM antibodies such as ELISA, lateral flow immunoassay-based Kits (LFIA) and chemiluminescence immunoassay-based Kits (CLIA) [32,33,34].

In a meta-analysis of COVID-19 serology tests that included 40 studies, the pooled sensitivity of IgG or IgM ELISA was 84.3% (95% confidence interval, 75.6% to 90.9%), for LFIAs it was 66.0% (49.3% to 79.3%), and for CLIAs it was 97.8% (46.2% to 100%). Among LFIAs, the pooled sensitivity of commercial kits was 65.0% (49.0% to 78.2%), which was lower than that of non-commercial tests, 88.2% (83.6% to 91.3%). 

Sensitivity was higher from three weeks after symptom onset (ranging from 69.9% to 98.9%) compared to within the first week (13.4% to 50.3%) [35]. The specificity of the evaluated assays was less variable and ranged between 96.6% to 99.7%. However, heterogeneity was observed in all analyses and methodological issues, such as patients’ selection bias, were identified across most of the studies included in this meta-analysis [36].

False-positive serological testing results have been associated with the presence of other viral infections (cytomegalovirus) or autoimmune diseases (such as scleroderma or systemic lupus erythematosus) [37,38].

### 2.4. Conventional Diagnostic Approaches

#### 2.4.1. Clinical Presentation

The most frequent symptoms of COVID-19 at presentation are cough (67.8%), fever (43.8%), and breathlessness (18.7%) [39]. Signs and symptoms in COVID-19 are however not specific and are often indistinguishable from those of other respiratory viruses. Even lack of smell or loss of taste are not specific for COVID-19 and can be easily confused with almost any other viral respiratory disease, which may also be endemic during winter period.

Clinical symptoms are not a reliable way of diagnosing COVID-19 patients except for patients with recent COVID-19 contact. Where there is significant transmission within a region or country, monitoring the number of healthcare consultations for COVID-19 compatible symptoms and establishing a diagnosis (including alternatives such as influenza and acute bacterial respiratory infection) helps identify further cases for testing (robust syndromic surveillance).

#### 2.4.2. Pulse Oximetry

Pulse oximetry is a simple and accessible tool for screening and continuous monitoring of patients with COVID-19. The target oxygen saturation range for patients with COVID-19 without comorbidities such as chronic obstructive pulmonary disease (COPD) is 92–96% [3,4,40]. The test is particularly useful in COVID-19 as patients can have severe hypoxemia in the absence of dyspnoea, sometimes referred to as “silent hypoxemia”. Home monitoring of patients with COVID-19, with patients only being admitted to hospital if the oxygen saturations decline below 92%, has been a valuable tool to reduce admissions and provide safe monitoring in the community [41,42]. Severe COVID-19 is suggested by oxygen saturation levels <92% (<88% in COPD) on room air, while in the absence of hypoxaemia most patients can be monitored at home [43].

Home SpO2 <92% was linked to a greater risk of intensive care unit admission, acute respiratory distress syndrome (ARDS) and septic shock. Therefore, a SpO2 cutoff of 92% was found to be a useful an indicator for readmission [44].

Initial oxygen saturation measured by ambulance staff in patients with COVID-19 patients correlate well with short-term (30-day) patient mortality or ICU admission. Even small deviations in oxygen saturation of 1–2% below 96% confer an increased mortality risk [45].

#### 2.4.3. Hematological Evaluation

Thrombocytopenia, leukopenia and neutropenia can all be seen in COVID-19 disease [39,46]. Lymphopenia (89.2%) is the most common finding on a full blood count (FBC), followed by neutrophilia (74.3%), and thrombocytopenia (24.3%) [47]. Lymphopenia and eosinopenia were also associated with severe disease and mortality and they were identified in 77.6% and 81.2% of non-survivors [48,49,50]. Although FBC patterns cannot be used for diagnosis alone, they can assist in triage and prognostication.

Several indices based on FBC variables have been developed to help guide COVID-19 diagnosis, prognostication and triage (Table 2). Systemic inflammation index (SII) is a biomarker predictive of in-hospital mortality and the development of ARDS [51].

Non-survivors had significantly higher index of systemic inflammation (AISI), derived neutrophil to lymphocyte ratio (dNLR), neutrophil to lymphocyte × platelet ratio (NLPR), neutrophil to lymphocyte ratio (NLR), systemic inflammation index (SII), and systemic inflammation response index (SIRI) values when compared to survivors. All of these indices are calculated based on FBC and their comparison is presented in Table 2. Kaplan–Meier survival curves showed significantly lower survival in patients with higher AISI, dNLR, MLR, NLPR, NLR, SII, and SIRI. However, after adjusting for confounders, SII was best at predicting survival [52].

Some of these inflammatory markers may also have a diagnostic value. Lymphopenia is suggestive of COVID-19 in patients with consistent clinical presentation and imaging. Moreover, it seems that most patients have a high NLR of >5 (94.5%), high SII index of >500 (89.2%), increased C-reactive protein (100%) and high level of IL6 (>10 pg/mL) [47].

Since the pathogenesis of COVID-19 is tightly linked to pro-inflammatory state and cytokine storm, several interleukins (IL) have been evaluated as diagnostic and prognostic markers. Baseline changes in IL-6, IL-10 and their ratio may be a good prognostic marker [53]. Studies have shown that IL-6 correlates with clinical outcomes, such as mortality and respiratory failure better than laboratory alternatives such as CRP, ferritin and liver enzymes [54,55]. A comparison of the inflammatory markers is presented in Table 3.

The concept of cytokine storm has been recently reappraised. A recent systematic review and meta-analysis based on 25 studies has reviewed the role of IL-6 in patients with severe or critical disease. Patients with COVID-19 (*n* = 1245 patients) were compared to patient with sepsis (*n* = 5320), cytokine release syndrome (*n* = 72), and acute respiratory distress syndrome unrelated to COVID-19 (*n* = 2767). Mean interleukin-6 concentrations were nearly 100 times greater in patients with cytokine release syndrome, 27 times higher in patients with septic shock, and 12 times higher in patients with ARDS unrelated to COVID-19 [58]. This data does not support the concept of a COVID-19-induced organ dysfunction.

Serum levels of IL-6 and IL-10 are also strong predictors of disease progression and severity [59]. Being inexpensive, they can be performed on arrival to hospitals or care centers with minimum facilities. Using these tests, earlier identification of patients at risk of severe disease may be possible.

#### 2.4.4. Coagulation

Coagulation dysfunction is a risk factor for adverse outcomes in COVID-19 and should be carefully considered in caring for patients [60]. Thrombocytopenia is detected in more than a half of the patients and its incidence varies according to disease severity. It is typically mild (100–150 × 10^9^/L) [61]. Although non-diagnostic, platelet counts and coagulation markers provide valuable information.

In one study, coagulation function in COVID-19 was assessed with thrombin-antithrombin complex (TAT), α2-plasmininhibitor-plasmin complex (PIC), thrombomodulin (TM), t-PA/PAI-1 complex (t-PAIC), prothrombin time (PT), international normalised ratio (INR), activated partial thromboplastin time (APTT), fibrinogen (FIB), thrombin time (TT), D-Dimer (DD), and platelet (PLT). The levels of TAT, PIC, TM, t-PAIC, PT, INR, FIB, and DD in COVID-19 patients were higher than healthy controls (*p* < 0.05) and in those with thrombotic disease than without thrombotic disease (*p* < 0.05). Patients with thrombotic disease had a higher case-fatality (*p* < 0.05). TAT, PIC, TM, t-PAIC, PT, INR, APTT, FIB, DD, and PLT also correlated with disease severity. T-PAIC and DD were independent risk factors for death and accurately predicted COVID-19 mortality risk [62,63,64]. Since a coagulation panel is a standard procedure, it is useful to assess several parameters that can indicate severity of the disease and a risk of thrombosis (Table 4).

#### 2.4.5. Comorbidities

Comorbid conditions often present a risk factor for adverse outcomes in infectious diseases. Numerous comorbidities have been correlated with adverse outcomes of COVID-19. However, COVID-19 outcomes are strongly correlated with age and gender, and most studies evaluating prognostic factors have not adequately accounted for these confounders. However, a recent meta-analysis using age and gender adjusted data confirmed strong associations between co-existing diseases such as chronic heart failure, ischemic heart disease, chronic obstructive pulmonary disease, diabetes mellitus, hypertension, malignancy, end-stage renal disease, dementia and obesity and increased COVID-19 related mortality [69].

A retrospective cohort study of 31,461 adults with COVID-19 from 24 healthcare organisations in the US demonstrated a high burden of comorbidities. Those most commonly listed were chronic obstructive pulmonary disease (COPD) (17.5%) and type 2 diabetes (15%). Using multivariate logistic regression analysis, higher odds of mortality with COVID-19 was seen across a range of conditions including dementia (OR 1.29), myocardial infarction (OR 1.97), cardiac failure (OR 1.42), COPD (OR 1.24), moderate to severe liver disease (OR 2.62), renal disease (OR 2.13) and metastatic malignancy (OR 1.70) [70]. This study highlights the need to have low clinical suspicion of COVID-19 in patients with multiple co-morbidities and to screen for moderate to severe disease early in the disease course.

Charlson comorbidity index (CCI), Elixhauser (ECI), and age- and smoking-adjusted Charlson (ASCCI) and Elixhauser (ASECI) comorbidity indices have also been used to predict COVID-19 outcomes. Patients with higher indices had a significantly longer time of hospitalisation (more than 24 days). CCI score >2 had a sensitivity of 79% and specificity of 71.9% for predicting longer time of hospitalisation, while ASCCI >3 had a sensitivity of 57.9% and specificity of 96.9%, and ASECI score >5 had a specificity of 57.9% and sensitivity of 90,6%. Using these scores, it may be possible to identify cases with potential for severe disease, and therefore a poorer prognosis earlier, allowing better treatment planning and communication [71].

Per point increase of CCI score is associated with an increased mortality risk of 16%. A higher mean CCI score is also significantly associated with mortality and disease severity. CCI score should be utilised for risk stratifications of hospitalised COVID-19 patients [72].

Vitamin D deficiency represents a well-established modifiable risk factor for severe COVID-19 disease and COVID-19 related mortality [73]. There is preliminary evidence suggesting that Vitamin D replacement could protect from COVID-19 contraction or adverse outcomes [73,74].

On the other hand, other comorbidities, such as asthma [75,76,77], urticaria and erythema nodosum [78] have been associated with decreased risk of adverse COVID-19 outcome. However, these diseases often affect younger people, who are anyway less prone to COVID-19 adverse outcomes, and therefore, the observed protective effect might be the result of confounding [69].

#### 2.4.6. Diagnostic Nomogram

Comorbid conditions often present a risk factor for adverse outcomes in infectious diseases. Numerous comorbidities have been correlated with adverse outcomes of COVID-19.

Several parameters such as older age, higher serum lactate dehydrogenase (LDH), CRP, coefficient of variation of red blood cell distribution width (RDW), direct bilirubin (DBIL), blood urea nitrogen (BUN), and lower albumin on admission have been correlated with higher odds of severe COVID-19. Therefore, they were used to elaborate a prognostic nomogram composed of seven features for early identification of patients at risk of exacerbation to severe COVID-19. The generated nomogram was efficient for early identification of severe COVID-19 in the training cohort (AUC 0.912 (95% CI 0.846–0.978), sensitivity 85.71%, specificity 87.58%); and in the validation cohort (0.853 (0.790–0.916), 77.5%, 78.4%) [79]. This diagnostic nomogram is assessed based on the admission laboratory tests.

#### 2.4.7. Imaging

A recent meta-analysis of 34 studies with 9339 patients with SARS-CoV-2 infection, confirmed using RT-PCR alone or RT-PCR with another test, assessed the diagnostic efficiency of chest X-ray (CXR), computed topography (CT) and ultrasound (USS). COVID-19 was correctly diagnosed based on CT thorax in 89.9% of patients with COVID-19 and incorrectly identified in 38% of patients who did not have COVID-19. The sensitivity of CXR ranged between 57–89%, while false-positive results ranged between 11–89% of patients without COVID-19. Finally, the sensitivity of lung ultrasound was 96%, while the corresponding false-positive rate was 38%. These findings demonstrate that chest imaging modalities are sensitive in identifying COVID-19 with lower respiratory tract involvement, while their specificity is limited [80]. Interestingly, inter-rater agreement on the interpretation of chest x-rays is moderate [81,82].

Pre-operative CT chest has been successfully and widely used as a screening tool for COVID-19 in people undergoing surgical procedures during the pandemic [83].

## 3. Conclusions

Molecular testing with RT-PCR is the gold-standard not only for screening and diagnosis of COVID-19, but also to follow-up disease progression. However, more affordable, direct antigen or indirect antibody COVID-19 tests, despite their more limited sensitivity, might be more cost-effective screening modalities. Selection of the optimal strategies for COVID-19 screening, diagnosis and prognostication amidst the pandemic is complex. Unfortunately, the vast costs associated with these processes during the pandemic limit equity globally and national strategies need to be informed by best available evidence, but also by economics. Prediction models based on simple laboratory tests, imaging and clinical variables can facilitate cost-effective prognostication and triage.

Further research is needed to develop accurate and affordable screening and diagnostic strategies, as well as accurate prognostic tools to facilitate identification of patients at risk of deterioration and resource allocation.

## Figures and Tables

**Table 1:** Comparison of molecular diagnostic tests.

Method	Biomarker	Description	Type of Clinical Sample	Operating Temperature	Assay Time	Advantages	Limitations
RT PCR	Nucleic acid (SARS-CoV-2 RNA)	– Regular real time PCR – Gold Standard	Respiratory specimens	Thermal cycling	2–4 h	– High performance characteristics	– Require equipment, resources, and expertise – Risk of cross contamination.
ddPCR	Nucleic acid (SARS-CoV-2 RNA)	– Droplet digital PCR – Limited dilution; probability theory-based analysis.	Throat swab	Thermal cycling	1 h	– High sensitivity in low viral load specimens	– Less affordable than RT-PCR. – Require specialised equipment and consumables.
RT-LAMP	Nucleic acid (SARS-CoV-2 RNA)	– Isothermal – Single step RNA reverse transcription and nucleic acid amplification.	Throat swab	30–65 °C	30 min	– High sensitivity and specificity.Uses simple instruments. – Does not require thermal cycling (and is therefore rapid and affordable). – Uses simple instruments.	– Challenges in generating sequence specific primers. However, commercially assays are now available. – An internal PCR inhibition control cannot be included, and therefore duplication of reactions is needed.
CRISPR (SHERLOCK)	Nucleic acid (SARS-CoV-2 RNA)	– CRISPR/Cas13 based nucleic acid detection method – Qualitative visual results.	Nasopharyngeal swabs		˂1 h	– Low cost – Higher sensitivity – Uses simple instruments.	– Cas13 does not catalytically activate when there are two or more mistakes in the crisper RNA: target duplex – Suboptimal quantification
CRISPR (DETECTR)	Nucleic acid (SARS-CoV-2 RNA)	– RT-LAMP – CRISPR Cas12-based lateral flow assay	Respiratory specimens	– C62 °C for RT LAMP – 37 °C for CRISPR-Cas12	˂40 min	– Rapid – Easy to implement – High sensitivity and specificity	– Risk of cross-contamination or suboptimal sample preparation, limiting accuracy.

**Table 2:** Inflammation indices related to mortality in COVID-19 [49].

Index	Formula	Optimal Cutoff Values and Performance Characteristics
SII	platelet × neutrophil/lymphocyte counts	Cut off point >1835 sensitivity 55% specificity 75%
SIRI	neutrophil × monocyte/lymphocyte	Cut off point >2.93 sensitivity 59% specificity 74%
AISI	neutrophil × platelet × monocyte/lymphocyte	Cut off point>798 sensitivity 59% specificity 72%
NLR	neutrophil/lymphocyte ratio	Cut off point >15.2 sensitivity 38% specificity 97%
NLPR	neutrophil/(lymphocyte × platelet)	Cut off point >0.019 sensitivity 66% specificity 75%
dNLR	neutrophils/(white blood cells -neutrophils)	Cut off point >6.2 sensitivity 52% specificity 85%

AISI—index of systemic inflammation, NLR—neutrophil to lymphocyte ratio, NLPR—neutrophil to lymphocyte × platelet ratio, NLR—neutrophil to lymphocyte ratio, SII—systemic inflammation index, SIRI—systemic inflammation response index.

**Table 3:** Inflammatory biomarkers.

Marker/Score	Applicability	Reference
CRP	Detection of severe/critical illness: Threshold: 41.4; Sensitivity: 90.5%; specificity: 77.6%; Positive predictive value: 61.3%; Negative predictive value: 95.4%	[56]
IL-6	In-hospital mortality: Threshold: 37.65 pg/mL; Sensitivity: 91.7%; Specificity: 95.7%. Rise in IL-6 from presentation to day 4 was predictive of a more severe clinical outcome (OR 1.14, 95% CI 1.07–1.21, per 10 units increase)	[53,57]
IL-6:IL-10 ratio	Rise in IL-6/IL-10 ratio from presentation to day 4 was predictive of increased risk of a more severe clinical outcome (OR 1.28, 95% CI 1.17–1.40, per 0.1 units increase)	[53]
Dublin-Boston score	Calculated by multiplying the day 0 to day 4 change in IL-6:IL-10 ratio by two, rounding to whole numbers, and then restricting the score to a 5-point scale ranging from −2 to 2. Rise in the Dublin-Boston score was predictive of a more severe outcome (OR 5.62, 95% CI −3.22–9.81, p = 1.2 × 10^−9^, per 1 point increase)	[53]

IL—interleukin, CRP—C-reactive peptide.

**Table 4:** Coagulation parameters helpful in COVID-19 triage.

Tests	Impact	Reference
DD	In-hospital mortality. Threshold: 2.0 µg/mL; Sensitivity: 92.3%; Specificity: 83.3%.	[63]
t-PAIC	In-hospital mortality. Threshold: 20.6 ng/mL; Sensitivity: 90.0%; Specificity: 91.2%.	[64]
PT	In-hospital mortality. Prolonged prothrombin time was predictive of mortality (OR: 2.19, 95% CI: 1.29-3.73).	[65]
FIB	Development of ARDS. Threshold: 617 mg/dL; Sensitivity: 76%; Specificity: 79%.	[66]
PLT	Mortality: Elevating platelet counts are predictive of decreased mortality. 50 × 10^9^/L increment increase in platelets was associated with 40% decrease in mortality. Among hospitalised patients, the platelet trajectory (increase or decrease) during hospital stay, was associated with decreased or increased mortality, respectively.	[67]
ISTH criteria of DIC	DIC is strongly associated with mortality. 71.4% of non-survivors fulfilled the ISTH criteria for overt DIC (≥5 points) in the later stages of coronavirus pneumonia, while the respective proportion among survivors was 0.6%.	[68]

TAT—thrombin-antithrombin complex, PIC—α2-plasmininhibitor-plasmin Complex, t-PAIC—t-PA/PAI-1 Complex, PT—prothrombin time, FIB—fibrinogen, DD—D-Dimer, PLT—platelet, ISTH—International Society on Thrombosis and Haemostasis, DIC—disseminated intravascular coagulation.

## Data Availability

Not applicable.
